# Draft Genome Sequence of *Tepidimonas taiwanensis* Strain MB2, a Chemolithotrophic Thermophile Isolated from a Hot Spring in Central India

**DOI:** 10.1128/genomeA.01723-15

**Published:** 2016-02-18

**Authors:** Darshan B. Dhakan, Rituja Saxena, Nikhil Chaudhary, Vineet K. Sharma

**Affiliations:** Metagenomics and Systems Biology Laboratory, Department of Biological Sciences, Indian Institute of Science Education and Research, Bhopal, Madhya Pradesh, India

## Abstract

*Tepidimonas taiwanensis* strain MB2 is a thermophile isolated from a hot spring located in central India. Here, we report a 2,849,160 bp draft genome sequence of *Tepidimonas taiwanensis* MB2. The genome shows properties of sulfur metabolism, nitrogen fixation, ammonia metabolism, assimilation of organic acids, and a wide variety of proteases.

## GENOME ANNOUNCEMENT

The microbes belonging to genus *Tepidimonas* are Gram-negative, strictly aerobic, thermophilic microbes from the phylum *Proteobacteria* (1, 2). The species identified to date were isolated from hot springs of moderately high temperatures (50 to 60°C) (3, 4). *Tepidimonas taiwanensis* strain MB2 reported in this study was isolated from a hot spring bore well of approximately 250-meters depth at Chhoti Anhoni, a hot spring located near Pachmarhi Hill station (22.65°N, 78.36°E) in the state of Madhya Pradesh, India. The on-site temperature, pH, and total dissolved solids at the collection site were recorded as 52°C, 7.8, and 620 ppm, respectively.

MB2 was isolated on tryptone yeast extract dextrose agar having a known composition (0.6% casein enzymatic hydrolysate, 0.3% yeast extract, 0.5% dextrose powder, 2% agar, and pH 7.5), supplemented with 0.125% dipotassium hydrogen phosphate. The cultures were incubated at 55°C under aerobic conditions and colonies were formed within 24 to 48 h of culture. The observed colonies were circular, medium-sized, brown, and smooth with an undulated margin. Gram staining showed Gram-negative rod-shaped bacteria. Genomic DNA was extracted from pure culture using the phenol-chloroform extraction methodology.

The 16SrRNA sequence of MB2 showed 100% sequence identity at 94% coverage with *Tepidimonas taiwanensis*. Genome sequencing of MB2 was performed on the Illumina NextSeq500 sequencing platform (Illumina, San Diego, USA) producing paired-end reads of 150-bp length. A total of 2.8 Gbp (fastq format) sequencing data was obtained and 4,911,330 high-quality reads were used for the assembly of the MB2 genome using SPAdes 3.5.0 (5). The draft genome size of MB2 is 28,49,160 bp in 230 contigs with an *N*_50_ contig length of 56,989 bp and an average contig length of 11,199.27 bp. The G+C content of MB2 was 68.91%. A total of 41 tRNAs and 2 rRNA operons were predicted by tRNAScan-SE version 1.3.1 and RNAmmer version 1.2, respectively (6, 7). A total of 2,503 protein coding genes were predicted using Glimmer version 3.02 (8). The genes were functionally annotated using BLAST (version 2.2.6) against the NCBI nr database and metabolic pathways were identified using KEGG automated annotation server (KAAS) (9–11). The genome contains *sox*A, *sox*X, and thiosulfate/3-mercaptopyruvate sulfurtransferase genes, which help in the oxidation of thiosulfate to sulfate revealing chemolithotrophic properties of the strain. The strain also has NADH dehydrogenase complex I and cytochrome *c* oxidase (complex IV) which shows its aerobic mode of respiration. The nitrogen regulatory proteins (P-II and P-II1) required for assimilation of nitrogen under low nitrogen conditions are present in the genome. The strain also contains genes for a wide range of proteases with potential industrial applications.

### Nucleotide sequence accession numbers.

This whole-genome shotgun project has been deposited at DDBJ/EMBL/GenBank under the accession no. LOQE00000000. The version described in this paper is version LOQE01000000.
